# Opportunistic Feeding and Parasite Assemblages in Sei Whales (*Balaenoptera borealis*) From Southern Chile Revealed by DNA Metabarcoding

**DOI:** 10.1002/ece3.74427

**Published:** 2026-09-28

**Authors:** Daniela Haro, Guillaume Schwob, Luis Bedriñana‐Romano, Valentina Bernal‐Durán, Benjamín Cáceres, Rodrigo Hucke‐Gaete, Miguel Cáceres, Olivia Blank, Camila Calderón‐Quirgas, Mauricio Ulloa, Francisco Viddi

**Affiliations:** ^1^ Centro Bahía Lomas, Facultad de Ciencias Universidad Santo Tomás Puerto Montt Chile; ^2^ Instituto Milenio Biodiversidad de Ecosistemas Antárticos y subantárticos (BASE) Santiago Chile; ^3^ UMR Ecologie Microbienne ‐ Lyon, CNRS 5557, INRAE 1418, Lyon 1 University, VetAgro Sup Université de Lyon Marcy L'Etoile France; ^4^ NGO Centro Ballena Azul Valdivia Chile; ^5^ Center for Oceanographic Research COPAS COASTAL Universidad de Concepción Concepción Chile; ^6^ Instituto de Ciencias Marinas y Limnológicas, Facultad de Ciencias Universidad Austral de Chile Valdivia Chile; ^7^ Departamento de Ciencias Básicas, Facultad de Ciencias Universidad Santo Tomás Puerto Montt Chile; ^8^ Asociación de Investigadores Museo de Historia Natural Río Seco Punta Arenas Chile; ^9^ Rewilding Chile Punta Arenas Chile; ^10^ Clínica Veterinaria Timaukel & Centro de Rehabilitación de Aves Leñadura Punta Arenas Chile; ^11^ Centro de Estudios de Mastozoología Marina Concepción Chile; ^12^ Servicio Nacional de Pesca y Acuicultura Iquique Chile; ^13^ Fundación Melimoyu Valdivia Chile

**Keywords:** *Balaenoptera borealis*, diet composition, DNA metabarcoding, *Grimothea gregaria*, parasites, southern South America

## Abstract

Balaenopterid whales are major consumers in pelagic ecosystems and play a fundamental role in marine food webs. The sei whale ( *Balaenoptera borealis* ) is a broadly generalist species that remains among the least studied baleen whales. In this study, we characterized diet composition and parasite assemblage in five stranded sei whales in southern Chile using DNA metabarcoding of stomach and intestinal contents. Four main prey categories were detected among the five individuals, with marked inter‐individual variations. *Grimothea* spp. occurred in all whales and dominated prey‐derived reads in the stomach contents (97.6%) with high values for the estimated occurrence indices (%FOO = 100, POO = 54.5 for 18S). Calanoid copepods (1.4%) represented the second most frequent prey group, occurring in three individuals. Myctophid fishes (0.6%), including *Lampanyctus parvicauda* and *Triphoturus oculeus* , previously unrecorded in the sei whale diet, were detected in two individuals. Euphausiids (0.4%) were detected in a single individual. Metabarcoding also detected DNA assigned to parasites, including trematodes, cestodes, and parasitic protozoans, in each whale examined. *Homalometron* spp. exhibited the highest read abundance across individuals, and the cestode genus *Tetrabothrius* was also consistently present. We additionally detected *Adenocephalus* spp., expanding the known host range of this genus to large baleen whales, and *Cryptosporidium* spp., which to our knowledge has not previously been reported in sei whales. Overall, our findings provide new insight into the trophic ecology of sei whales foraging in southern Chile and reinforce the ecological importance of *Grimothea* spp. as a key prey resource in austral marine ecosystems. This study also highlights the potential of DNA metabarcoding to recover both dietary and parasite‐associated signals from rare necropsy material in an endangered whale species.

## Graphical abstract

This study represents the first metabarcoding‐based description of prey composition and parasite‐associated taxa in stranded sei whales from southern South America.

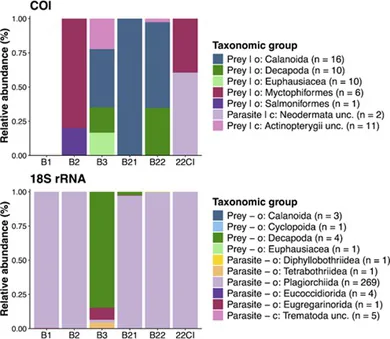

## 1 Introduction

Characterizing animal diet is fundamental for understanding ecological function, habitat use, foraging strategies and species interactions (Pierce and Boyle 1991; Bowen and Iverson 2013; Zhang et al. 2023). Moreover, dietary information is also essential for evaluating trophic roles anticipating responses to environmental change, and informing conservation strategies of both species and ecosystems (Parrish et al. 2002; Bowen and Iverson 2013). In parallel, documenting parasite assemblages on wildlife has been pinpointed as a major conservation concern due to the risk of zoonosis and how emergent pathogens relate to habitat modification and climate change (Van Bressem et al. 2009; Jenkins et al. 2015). In this context, cetaceans are of particular interest because they harbor a wide diversity of endo‐ and ectoparasites in a variety of tissues and organs (Dailey 2001).

Balaenopterid whales are among the largest consumers in the ocean and play an important role in pelagic food webs and biogeochemical processes (Roman and McCarthy 2010; Roman et al. 2014; Savoca et al. 2021). Their diets generally include small crustaceans from the zooplankton, squids, and small fishes (Kawamura 1980; Barros and Clarke 2002), with variations depending on geographic location (Tomilin 1967). However, direct observation of feeding in cetaceans is logistically difficult, and dietary reconstruction has traditionally relied on stomach, intestinal, and fecal content analyses, which involve the recovering and identification of resistant parts of consumed prey in the digestive material (Chittleborough 1965; Barros and Clarke 2002). Alternatively, or complementarily, diet determination is often carried out using chemical approaches, such as stable isotope analysis of carbon and nitrogen (Newsome et al. 2010; Haro et al. 2021), quantitative fatty acid signature analysis (Budge et al. 2006), and DNA analysis (De Vos et al. 2018; Reidy et al. 2022). In particular, diet analyses have benefited from the application of DNA metabarcoding on stomach or fecal samples, complementing the information provided by traditional methods (Pompanon et al. 2012; Myers et al. 2025). DNA metabarcoding combines the amplification of standardized genetic markers using broadly targeted primers with high‐throughput sequencing and comparison against reference databases, allowing multiple prey taxa to be identified simultaneously. Unlike morphological analyses, which rely on recognizable prey remains, or chemical approaches, which generally provide indirect information on broad dietary sources or trophic position, metabarcoding can identify digested or otherwise visually unrecognizable prey at relatively fine taxonomic resolution. It can also rapidly process large numbers of samples (Hebert and Gregory 2005), and describe local aquatic biodiversity based on dietary DNA (i.e., DNA extracted from intestinal, stomach, or fecal contents) (de Sousa et al. 2019). In addition to diet, metabarcoding allows the simultaneous assessment of populations genetics, parasites, and gut microbiota (Srivathsan et al. 2016; Cabodevilla et al. 2023) which has allowed a detailed parasitological inspection on free ranging baleen whales (Hermosilla et al. 2015).

Among baleen whales, the sei whale ( *Balaenoptera borealis* ) is one of the least known species and is currently listed as Endangered on the IUCN Red List of Threatened Species (Cooke 2018). They are distributed across all ocean basins from temperate waters to high latitudes; however, unlike other baleen whales, they do not regularly extend into polar or tropical regions (Mizroch et al. 1984; Horwood 2009). Three populations with significant genetic divergence have been described for this species: North Atlantic, North Pacific, and Southern Hemisphere (Rice 1998; Pérez‐Álvarez et al. 2021). Sei whales are generally considered trophic generalists, and in the Southern Hemisphere, they have been reported to consume copepods, euphausiids, amphipods, squat lobster, and, to a lesser extent, squids and small fish (Mikheyev 1965; Nemoto and Kawamura 1977; Budylenko 1978; Acevedo et al. 2017; Buchan et al. 2021; Weir and Prieto 2024). Closely related to their feeding habits and even more underassessed, the diversity of organisms known to parasitize on sei whales globally consists of 16 species of helminth; the most common being the acanthocephalan *Bolbosoma turbinella* , the nematode *Anisakis simplex*, and the cestode *Tetrabothrius* (Hermosilla et al. 2015; Leonardi et al. 2011; Matthews 1938; Weir and Prieto 2024).

In Chile, over the past decades, sei whales have been recorded, mainly in northern Patagonia (41°–42° S; Hucke‐Gaete et al. 2006; Galletti‐Vernazzani et al. 2006) (46°–48° S, Español‐Jiménez et al. 2019), around the Magellan Strait (53°–54° S; Acevedo et al. 2017), and in the central region (36° S; Cisterna‐Concha et al. 2023). Likewise, several strandings have been reported, primarily in the Chilean Patagonia (Häussermann et al. 2017; Schüttler et al. 2024). Despite these records, there is limited information regarding the sei whale trophic habits (Acevedo et al. 2017; Buchan et al. 2021), and there are no records of endoparasites for this population. There is only one report of feeding habits through direct observation, indicating the consumption of sardine ( *Sprattus fuegensis* ) in the Strait of Magellan (Acevedo et al. 2017), and a study with analysis of four sei whale feces that indicates the predominance of squat lobster (*Grimothea gregaria*, Fabricius 1793, formerly *Munida gregaria*) in its diet (Buchan et al. 2021). Considering the critical relevance of basic biological information, such as feeding habits and parasite diversity, for the effective conservation of an endangered species, in this study we used DNA metabarcoding to (1) assess diet composition and (2) describe the presence of parasites in the stomachs and intestines of five stranded sei whales from southern Chile. Based on reports on the diet of this species in the Southern Hemisphere and the two records of their diet in Chile (Acevedo et al. 2017; Buchan et al. 2021), we predict that sei whales in southern Chile feed on both zooplankton and small fish, with *Grimothea gregaria* and *Sprattus fuegensis* being the predominant prey species.

## 2 Methods

### 2.1 Sample Collection

Samples were collected from seven stranded sei whales in southern Chile under Sernapesca sampling permits no. 2022‐70‐2 and no. 2015‐11‐01 (Hucke‐Gaete et al. 2015; Häussermann et al. 2017; Zegers et al. 2020; Caceres et al. 2022; Schüttler et al. 2024) (Figure 1, Table 1). While the sample size is limited, stranded individuals provide rare access to stomach contents in large baleen whales, and metabarcoding allows high‐resolution dietary reconstruction from otherwise inaccessible data. Prior to sample collection, the total body length of each individual was measured using a measuring tape with centimeter precision. Sex was determined visually by the presence of a penile protrusion or mammary slit openings. Carcass decomposition stage was assessed following Geraci and Lounsbury (2005). Sexual maturity was estimated from body length, considering that females reach maturity at approximately 14 m and males between 13 and 14 m (Best and Lockyer 2002; Horwood 2009).

**FIGURE 1:**
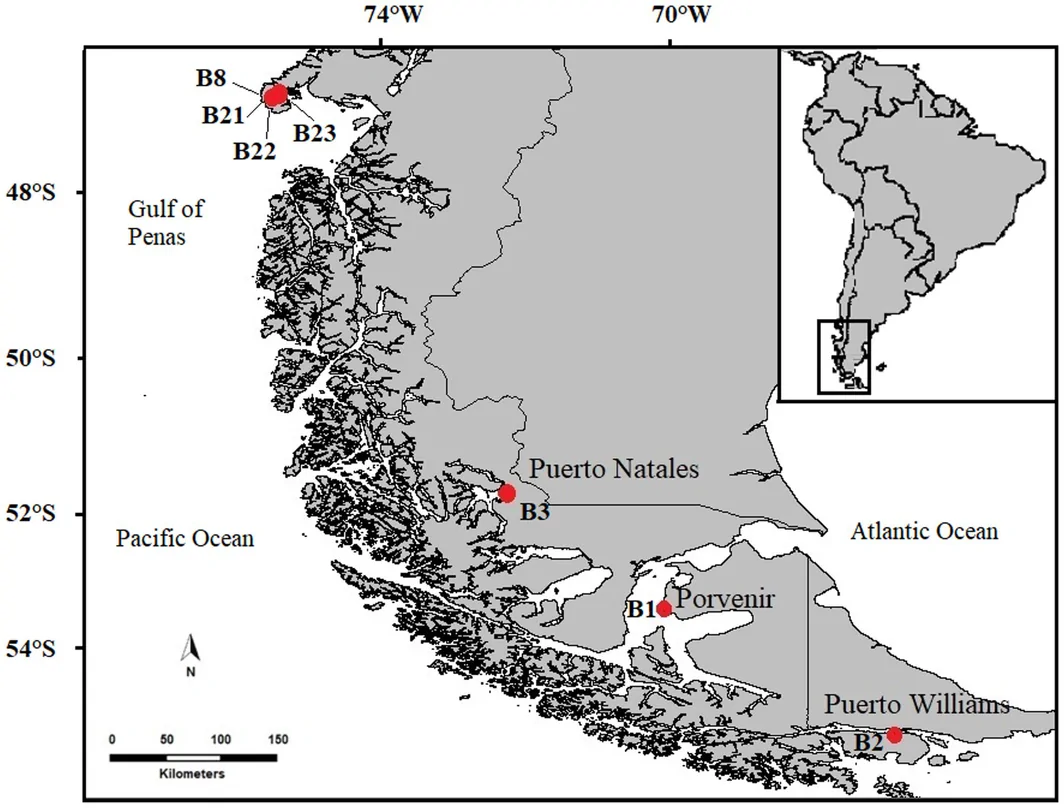
Sampling site for stranded sei whale individuals in southern Chile.

**TABLE 1:** Biological information stranded *Balaenoptera borealis* (sei whale) individuals and their corresponding stomach content samples collected in southern Chile.

Sample ID	Sex	Body length (m)	Age class	Date of death	Sampling date	Descomposition stage	Site	Sample type	Sample volume (mL)	Subsample volume (mL)	Extracted mass (mg)	DNA conc. (ng/μL)
B08	Female	15.3*	Adult	−/02–04/2015	27/05/2015	4	Gulf of Penas	Stomach content	500	15	300	0
B21	Male	8.3	Juvenile	−/02–04/2015	27/05/2015	4	Gulf of Penas	Stomach content	500	15	340	0
B22	Female	10.0	Juvenile	−/02–04/2015	27/05/2015	4	Gulf of Penas	Stomach content	250	15	311	17.9
								Intestinal content	500	15	316	39.4
B23	Male	11.5	Juvenile	−/02–04/2015	27/05/2015	4	Gulf of Penas	Stomach content	400	15	298	0.4
B01	Female	15.7	Adult	08/03/2020	10/03/2020	3	Porvenir, Tierra del Fuego Island	Intestinal content	2500	30	300	8.9
B02	Female	15.2	Adult	09/03/2020	14/03/2020	3	Puerto Williams, Navarino Island	Stomach content	3000	45	295	3.6
B03	Male	10.4	Juvenile	28/04/2022	04/05/2022	3	Puerto Natales	Intestinal content	500	15	299	4.3

*Note:* Decomposition stages follow categories: Stage 3 (Moderate decomposition), carcass generally intact, mild abdominal distension, protrusion of tongue and penis, localized skin sloughing, internal organs grossly intact, and eyes sunken or absent; Stage 4 (Advanced decomposition), carcass may be intact but collapsed with evident abdominal distension, areas devoid of epidermis and skin, internal organs lacking structural integrity, and scavenger severe damage.

* indicate the approximate measurement.

Partial necropsies were performed to recover stomach and/or intestinal contents from the stranded whales (Hucke‐Gaete et al. 2015; Zegers et al. 2020; Caceres et al. 2022) following the procedures described by Geraci and Lounsbury (2005). In total, eight digestive‐content samples were obtained, including five stomach samples and three intestinal samples from seven individuals (Table 1). Five samples (i.e., individuals B8, B21, B22, and B23; Table 1) were obtained during an expedition conducted in 2015 in the Gulf of Penas (46°46′ S; 75°32′ W) led by the Chilean Navy and the National Fisheries and Aquaculture Service. These samples were stored in the laboratory of the Blue Whale Center at the Universidad Austral de Chile (Hucke‐Gaete et al. 2015). The date of death of these individuals was estimated to have most likely occurred between February and April 2015 (Häussermann et al. 2017). Three of the analyzed samples correspond to specimens from the collection of the Río Seco Natural History Museum (i.e., individuals B1, B2, and B3; Table 1). Original sample volumes ranged between 250 and 3000 mL. After collection, samples were preserved in ethanol and frozen at −20°C until laboratory analysis. For molecular analysis, one subsample (15–45 mL) was taken from each stomach or intestinal content sample by combining small portions randomly collected from different areas of the original sample.

### 2.2 Genomic DNA Extraction

In the laboratory, each 15–45 mL composite subsample was briefly vortexed to homogenize its contents. Approximately 300 mg of homogenized material was targeted for each extraction. However, because the stomach and intestinal contents occasionally contained small solid fragments, the exact mass varied slightly among samples, ranging from 290 to 340 mg (Table 1). Genomic DNA was extracted using the DNEasy PowerSoil Pro kit (Qiagen), following the manufacturer's protocol with slight modifications. These include a preliminary lysis step that included a 10‐min incubation at 65°C, followed by mechanical homogenization at 4.0 m/s for 20 s, using a FastPrep‐24 bead beating grinder (MP Biomedicals). DNA concentration and purity were measured using a NanoDrop spectrophotometer (Thermo Fisher Scientific), and extracted DNA was stored at −20°C until further analysis.

### 2.3 DNA Metabarcoding Library Preparation and Sequencing

Metazoans amplicon libraries were generated by Australomics (Chile). All DNA samples were amplified using two complementary DNA barcode regions selected to maximize prey detection and taxonomic coverage. First, a ≃ 313‐bp fragment of the mitochondrial cytochrome c oxidase subunit I gene (COI) was amplified using the primers mlCOIintF (Leray et al. 2013) and jgHCO2198 (Geller et al. 2013). COI was selected because its relatively high interspecific variability provides fine taxonomic resolution among metazoans (Leray et al. 2013). Second, a 300–500‐bp fragment of the nuclear 18S rRNA gene was amplified using the primers SSUF04 (Blaxter et al. 1998) and SSURmod (Sinniger et al. 2016; Günther et al. 2022). The 18S rRNA gene was selected because its conserved primer‐binding regions allow broad amplification across phylogenetically diverse eukaryotic and metazoan lineages (Hadziavdic et al. 2014). Thus, the combined use of COI and 18S was intended to provide complementary taxonomic resolution and coverage for characterizing metazoan prey diversity in whale digestive contents. Library preparation and sequencing were carried out using the Labex GenSeq platform (University of Montpellier, France). Libraries were prepared using the Nextera XT Index Kit (Illumina, Hayward, CA, USA) in separate reactions for each marker. PhiX control was added for quality calibration. Sequencing was performed on an Illumina MiSeq platform using a paired‐end sequencing kit (2 × 250 bp).

### 2.4 Bioinformatic Analysis

Raw amplicon sequences were demultiplexed using Cutadapt v2.10 (Martin 2011) in a UNIX environment. Demultiplexed reads were processed through a quality control pipeline combining Trimmomatic v0.39 (Bolger et al. 2014) and PRINSEQ v0.20.4 (Schmieder and Edwards 2011) to remove Illumina adapter sequences, trim bases with low quality (Phred score < Q30), and filter out reads with average Phred quality score (≥ Q30) or containing ambiguous bases. High‐quality reads were then imported in R version 4.5.2 (Team, R Core 2020) for amplicon sequence variant (ASV) inference using the DADA2 package (Callahan et al. 2016), following standard guidelines. Briefly, sequencing error rates were learned from dereplicated reads, and paired‐end reads were merged into ASVs based on error‐corrected models. Chimeric sequences were removed using the consensus method implemented in the DADA2 package. Taxonomic assignment of ASVs was performed in DADA2 using the Naive Bayesian Classifier method, against curated COI and 18S MetaZooGene databases (Mode‐C) from the South Pacific and South Atlantic Oceans (https://metazoogene.org/mzgdb/; Bucklin et al. 2021). Reference sequences containing ambiguous nucleotides were removed prior to assignment. A minimum bootstrap confidence threshold of 50% was applied to retain taxonomic annotations (Tables S1 and S2). Potential contaminant ASVs were identified using the decontam R package (Davis et al. 2018) with the prevalence method and were subsequently filtered out, along with singleton ASVs (ASVs with read counts < 2).

### 2.5 Diversity Analyses

Rarefaction curves were generated for each sample and for both markers using the non‐rarefied ASV contingency tables and the *ggrarecurve* function implemented in the MicrobiotaProcess R package (Xu et al. 2023). The COI and 18S rRNA contingency tables were transformed into relative abundances to account for varying sequencing depth and were subsequently aggregated at order, family, and genus levels for taxonomic visualization. Host‐derived ASVs were removed prior to visualization to avoid graphical dominance by host sequences and to improve the interpretability of the prey and parasite community patterns.

Prey were identified based on previously reported dietary components of sei whales, including copepods, euphausiids, amphipods, squat lobsters, squids, and small fish (Mikheyev 1965; Nemoto and Kawamura 1977; Budylenko 1978; Acevedo et al. 2017; Buchan et al. 2021; Weir and Prieto 2024). The frequency of occurrence (FOO), corresponding to the number of samples containing prey item *i*, was calculated. These values were expressed as a percentage (%FOO) according to:

$$\%{\mathrm{FOO}}_{i}=\frac{1}{S}\;\sum_{k=1}^{S}I_{i,k}\times 100\%$$

The relative percentage of occurrence (POO) was estimated for each prey item according to:

$${\mathrm{POO}}_{i}=\frac{\sum_{k=1}^{S}I_{i,k}}{\sum_{i=1}^{T}\sum_{k=1}^{S}I_{i,k}}$$

where *S* is the number of samples, *I* is an indicator function such that *I*_*i,k*_ = 1 if prey item *i* is present in sample *k*, and 0 otherwise, and *T* corresponds to the total number of prey items. POO represents %FOO rescaled so that the sum across all prey items equals 100%. POO provides a clear representation of the contribution of each prey item as a percentage of the total diet (Deagle et al. 2019). All analyses were performed using R.

## 3 Results

### 3.1 Sequencing and Data Processing

DNA was successfully extracted from six of the eight collected digestive‐content samples for metabarcoding analyses. After quality filtering and bioinformatic processing of the COI and 18S amplicon sequences, a total of 2,167,498 and 2,181,103 reads were retained, respectively. Following removal of host‐derived sequences, the final datasets used for downstream analyses comprised 609,393 reads (mean reads per sample = 101,566 ± 28,325; 1405 ASVs) for COI and 1,463,415 reads (mean reads per sample = 243,903 ± 34,085; 466 ASVs) for 18S, which were used for subsequent analyses (Tables S3 and S4). Rarefaction curves reached saturation for most samples, particularly for stomach contents, indicating adequate sequencing depth to capture the dominant taxonomic signal in the samples (Figure S1).

### 3.2 Molecular Analysis of Diet Composition

All samples contained organisms belonging to the phyla Rotifera, Cnidaria, and Platyhelminthes, as well as organisms from the class Ascidiacea and the genus *Grimothea*, protozoans from the phyla Apicomplexa and Ciliophora, and green algae (Chlorophyta) (Tables S3 and S4).

COI results indicated that sei whale DNA was present in all six analyzed samples (Table S3), representing 91% of the reads of the amplicon. The analysis identified 11 distinct phyla: nine of invertebrates, Chordata, and one algal phylum (Rhodophyta) (Table S3). The most abundant ASVs corresponded to Cnidaria (*n* = 79), Arthropoda (*n* = 47), and Rotifera (*n* = 38). Among the prey categories detected, COI metabarcoding revealed crustaceans from the class Malacostraca (*n* = 1268 reads, 36.1% of prey reads from that amplicon), represented by the genera *Grimothea* and *Euphausia*, and the class Copepoda (*n* = 1142 reads, 32.5% of prey reads from that amplicon), represented by the genera *Calanus*, *Subeucalanus*, and *Eucalanus*, as well as fishes from the class Actinopterygii (*n* = 1103 reads, 31.4% of prey reads from that amplicon), including myctophids of the genera *Triphoturus* and *Lampanyctus* (Figure 2, Table 2). In terms of frequency of occurrence, the class Malacostraca was the most frequently detected prey group (%FOO = 66.7 and POO = 22.2), followed by the family Subeucalanidae (%FOO = 50.0 and POO = 16.6) (Table 2).

**FIGURE 2:**
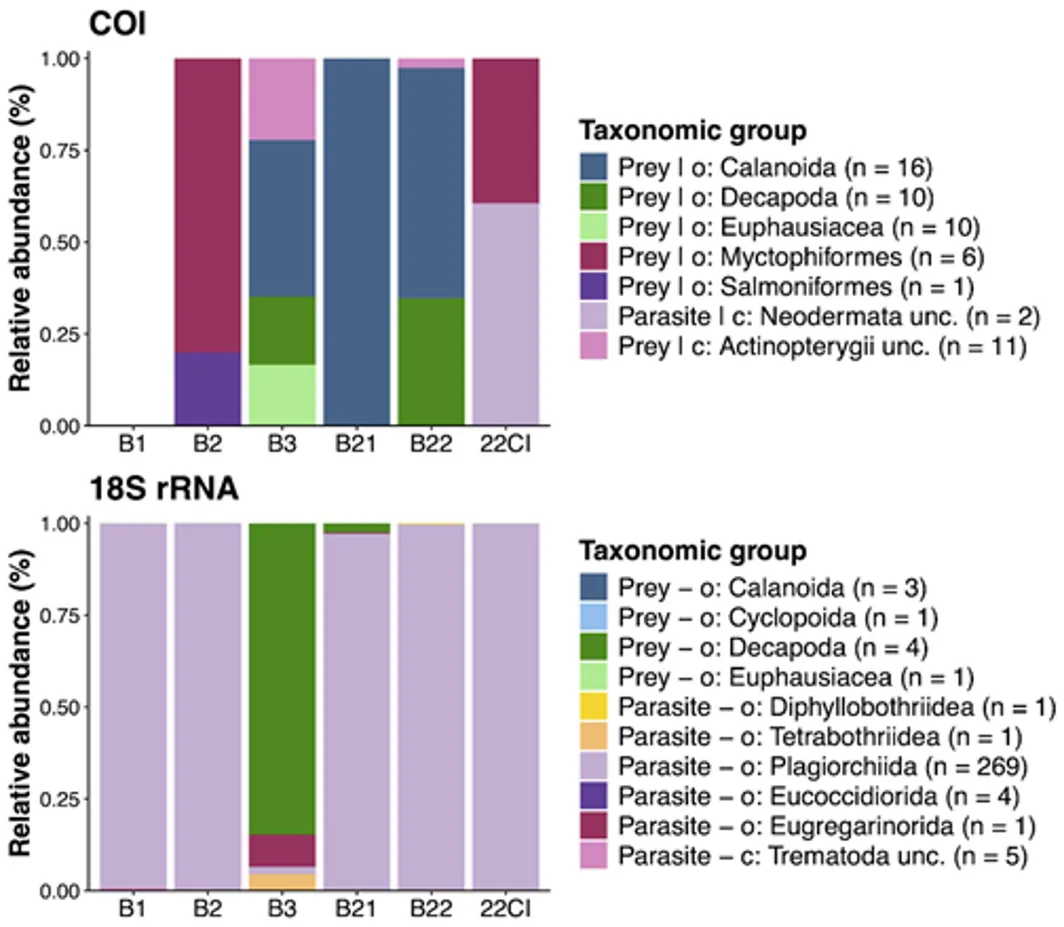
Relative abundance of prey and parasites found in sei whales analyzed with the COI and 18S amplicons. The number of ASVs corresponding to each taxonomic group is indicated in parentheses. Taxonomic abbreviations: c, class; O, order; Unc, unclassified.

**TABLE 2:** Number of prey and parasite taxon reads detected using COI and 18S amplicons in five stranded sei whales from southern Chile.

Category prey	B21	B22	IC B22	B1	B2	B3	Total	%FOO	POO
**COI**									
**Class Copepoda**	—	—	—	—	—	—	—		
**Order Calanoida**	0	107	0	0	0	0	107	16.7	5.6
Calanidae (*Calanus chilensis*)	0	0	0	0	534	0	534	16.7	5.6
Subeucalanidae	110	186	0	0	95	0	391	50.0	16.6
Eucalanidae	110	0	0	0	0	0	110	16.7	5.6
**Class Malacostraca**	0	98	106	0	186	64	454	66.7	22.2
**Order Decapoda**	—	—	—	—	—	—	—		
Munididae (Grimothea *gregaria*)	0	162	0	0	344	0	506	33.3	11
**Order Euphausia**	—	—	—	—	—	—	—		
Euphausiidae	0	0	0	0	308	0	308	16.7	5.6
**Class Actinopterygii**	0	12	0	0	440	0	452	33.3	11
**Order Myctophiformes**	—	—	—	—	—	—	—		
Myctophidae ( *Lampanyctus parvicauda* )	0	0	0	0	373	0	373	16.7	5.6
Myctophidae ( *Triphoturus oculeus* )	0	0	185	0	0	0	185	16.7	5.6
**Order Salmoniformes**	—	—	—	—	—	—	—		
Salmonidae	0	0	0	0	93	0	93	16.7	5.6
**Parasite**									
**Phylum Platyhelminthes**	0	0	284	0	0	0	284	16.7	100
**18S**									
**Class Copepoda**	—	—	—	—	—	—	—		
**Order Calanoida**	—	—	—	—	—	—	—		
Calanidae	0	0	0	0	61	0	61	16.7	9.1
Metridinidae	0	0	0	0	18	0	18	16.7	9.1
Clausocalanidae	0	0	0	0	13	0	13	16.7	9.1
**Order Cyclopoida**	—	—	—	—	—	—	—		
Oithonidae	0	0	0	0	8	0	8	16.7	9.1
**Class Malacostraca**	—	—	—	—	—	—	—		
**Order Decapoda**	—	—	—	—	—	—	—		
Munididae	199	144	102	134	86,695	129	87,403	100	54.5
**Order Euphausia**	—	—	—	—	—	—	—		
Euphausiidae	0	0	0	0	6	0	6	16.7	9.1

Parasite	B21	B22	IC B22	B1	B2	B3	Total	%FOO	POO
**Class Trematoda**	0	0	0	1790	0	49	1839	33.3	5.5
**Order Plagiorchiida**	1594	5781	8410	152,136	765	257,204	425,850	100	16.7
Apocreadiidae	0	16	0	1165	0	135	1316	50	8.3
Apocreadiidae (Homalometron)	6219	212,962	288,534	67,102	1240	8380	584,437	100	16.7
**Class Cestoda**	—	—	—	—	—	—	—		
**Order Tetrabothriidea**	—	—	—	—	—	—	—		
Tetrabothriidae (Tetrabothrius)	9	7	11	206	4644	9	4886	100	16.7
**Order Diphyllobothriidea**	—	—	—	—	—	—	—		
Diphyllobothriidae (Adenocephalus)	0	553	97	4	4	0	658	66.7	11.1
**Class Conoidasida**	—	—	—	—	—	—	—		
**Order Eugregarinorida**	—	—	—	—	—	—	—		
Lecudinidae	28	8	21	10	8954	15	9036	100	16.7
Cryptosporidiidae (Cryptosporidium)	0	0	4	0	0	35	39	33.3	5.5
Eimeriidae	0	0	0	0	0	27	27	16.7	2.8

Abbreviation: IC, Intestinal content.

The 18S dataset revealed 22 different phyla: six of invertebrates, Chordata, five of Chromista, eight of Protozoa, and two of Plantae (Table S4). The most abundant ASVs corresponded to Platyhelminthes (*n* = 278) and Protozoa (*n* = 66) (Table S4). Prey detected with this amplicon included crustaceans from the classes Malacostraca (*n* = 87,409 reads, 99.9% of prey reads from that amplicon) and Copepoda (*n* = 100 reads, 0.1% of prey reads from that amplicon), with the genus *Grimothea* being dominant (87,403 reads, 99.9% of prey reads from that amplicon) (Table S4, Table 2). In terms of frequency of occurrence, the family Munididae was the most frequently detected prey taxon (%FOO = 100 and POO = 54.5) (Table 2). The 18S amplicon also indicated the presence of parasitic worms belonging to the classes Trematoda and Cestoda (Table 2), as well as parasitic protozoans (class Conoidasida) from the families Lecudinidae, Cryptosporidiidae, and Eimeriidae (Table 2).

### 3.3 Individual‐Level Patterns

The prey found in the stomach contents of individual B21 (juvenile male) consisted of calanoid copepods from the families Subeucalanidae (26.3% of prey reads) and Eucalanidae (26.3% of prey reads), and squat lobsters from the family Munididae (47.4% of prey reads). Regarding parasites, trematodes belonging to the genus *Homalometron* were predominant, representing 79.2% of parasite reads (Table 2, Figure 2). In individual B22 (juvenile female), the analysis of stomach and intestinal contents revealed the presence of calanoid copepods from the family Subeucalanidae (16.9% of prey reads), squat lobsters *Grimothea gregaria* (37% of prey reads), and myctophid fish *Triphoturus oculeum* (16.8% of prey reads). 97.1% of the parasites detected in this individual corresponded to trematodes of the genus *Homalometron* (Table 2, Figure 2).

In the sei whale B1 (adult female) stranded in Porvenir (Figure 1), 100% of the prey identified belonged to the family Munididae. Among the parasites, 99% were trematodes belonging to the order Plagiorchiida (68.4% of parasite reads) and the genus *Homalometron* (31.2% of parasite reads) (Table 2). In the adult female stranded in Puerto Williams (B2, Figure 1), 97% of the prey corresponded to the family Munididae. The remaining prey included fishes from the families Myctophidae and Salmonidae (1% of prey reads), calanoid and cyclopoid copepods (0.8% of prey reads), and euphausiids (0.4% of prey reads). Parasites identified in this individual included protozoan from the family Lecudinidae (57.4% of parasite reads), cestodes of the genus *Tetrabothrius* (30% of parasite reads), and trematodes of the genus *Homalometron* (8% of parasite reads) (Table 2). In individual B3 (juvenile male) stranded in Puerto Natales (Figure 2), crustaceans from the class Malacostraca were identified, with the family Munididae being dominant (67% of prey reads). Trematode worms represented 99% of the parasites detected in this whale (Table 2, Figure 2).

## 4 Discussion

Accurate identification of the diet of top marine predators is essential for understanding trophic interactions, ecosystem functioning, and potential food‐web responses to environmental change (Moore and Huntington 2008; Simmonds and Isaac 2007). In cetaceans, however, diet assessment is particularly challenging because they spend most of their lives underwater, limiting direct observations of feeding behavior, and due to the complexity of the marine environment and the persistent difficulty in obtaining adequate sample sizes (Zhang et al. 2023; Reidy et al. 2022). Consequently, the development of robust techniques capable of providing reliable dietary information from limited cetacean samples has become an urgent priority for advancing their ecological study and informing conservation efforts (Reidy et al. 2022). Here, we provide the first metabarcoding‐based description of prey composition and parasite‐associated taxa in five sei whales stranded in southern Chile.

### 4.1 Molecular Analysis of Diet Composition

Several taxa detected in the digestive contents, including protozoans, rotifers, cnidarians, ascidians, and green algae, are unlikely to represent primary prey but instead likely reflect incidental ingestion of organisms attached to prey or suspended in the water column during bulk filter feeding. Similar non‐target detections were reported in metabarcoding analysis of sei whale feces, revealing not only expected prey taxa but also cnidarians and tunicates (Appendicularia) (Jackson et al. 2022), consistent with the balaenopterid feeding mode involving the engulfment of large volumes of seawater (Werth 2001; Goldbogen et al. 2017). For humpback whale ( *Megaptera novaeangliae* ) fecal samples, Reidy et al. (2022) referred to secondary consumption to describe a low‐abundance mixture of sequences that may correspond to the stomach contents of the whales' prey. More broadly, these results also illustrate that metabarcoding approach on stomach and intestinal contents can recover both trophic and surrounding biodiversity signals (Boyer et al. 2015; Siegenthaler et al. 2019). Despite inherent methodological biases, the concept of “dietary DNA” (dDNA) has been proposed to describe eDNA‐based approaches using gut‐derived material to characterize local biodiversity (de Sousa et al. 2019). This dual capacity to resolve both trophic and environmental contexts is now well established across biological domains, including microbial communities, as illustrated by metabarcoding studies of Patagonian teleost intestines revealing fine‐scale coupling between host diet, resident microbiota, and surrounding microbial diversity (Schwob et al. 2024).

Based on prey previously reported in the diet of sei whales, we identified prey consumed by five individuals in this study. Our results suggest that sei whales in Southern Chile exploit a diverse prey spectrum, including squat lobsters, copepods, myctophid fishes, and euphausiids. Among these, *Grimothea* spp. overwhelmingly dominated the prey signal, detected in all individuals at high read abundance and high values for the estimated occurrence indices. This result is consistent with previous records of *G. gregaria* in the stomach contents of sei whales caught in Patagonia (Matthews 1932). In the Falkland/Malvinas Islands, sei whales have been observed feeding on *G. gregaria* and *Themisto gaudichaudii* (Weir 2022; Weir and Prieto 2024). Similarly, Buchan et al. (2021) documented *G. gregaria* in sei whale feces collected in southern Chile. Our results reinforce the central role of *Grimothea* spp. in the diet of sei whales and highlight its broader ecological importance for balaenopterid foraging in southern Chile (Acevedo et al. 2011; Haro et al. 2021, 2023). In coastal ecosystems of southern South America, *G. gregaria* forms dense aggregations and may fulfill a functional role analogous to that of Antarctic krill ( *Euphausia superba* ) (Antezana 1999; Tapella et al. 2002). Its strong dominance in our dataset likely reflects predictable aggregation dynamics in fjord and channel systems, suggesting that sei whale foraging is closely linked to mesoscale patterns of productivity in these environments.

Calanoid copepods were the second most abundant and prevalent prey in the analyzed sei whale individuals, consistent with their well‐established role as a dominant dietary component and with the species' baleen morphology, which enables efficient feeding on small, low‐density zooplankton while retaining the capacity to engulf larger or more dispersed prey (Mizroch et al. 1984; Horwood 2009; Brodie and Vikingsson 2009). Across Antarctic, Subantarctic, and South Atlantic waters (30°–50° S), sei whales are known to consume calanoid copepods, particularly *Calanus tonsus* and *C. simillimus* (Kawamura 1974; Budylenko 1978; Jackson et al. 2022; Weir and Prieto 2024), with additional evidence from Chile indicating a broader diet that includes euphausiids and amphipods (Budylenko 1978; Reiss et al. 2020). In this study, calanoid copepods were represented by five families (Calanidae, Subecalanidae, Eucalanidae, Metridinidae, and Clausocalanidae), alongside cyclopoid copepods of the family Oithonidae. This taxonomic composition is consistent with previous reports from the Southern Hemisphere, where similar calanoid families have been identified as predominant in sei whale feces from the Falkland/Malvinas Islands (Jackson et al. 2022). In contrast, the detection of cyclopoid copepods (family Oithonidae) in the Puerto Williams individual likely reflects incidental ingestion, given their small size (0.4–1.4 mm; Böttger‐Schnack 1988), low energetic value, and reduced swarm size (Jackson et al. 2022).

A third prey category reported in this study was myctophid fishes, including *Lampanyctus parvicauda* in the Puerto Williams individual and *Triphoturus oculeus* in a whale stranded in the Gulf of Penas. Sei whales are known to feed on small schooling fishes (Mizroch et al. 1984; Burkhardt‐Holm and N'guyen 2019). For instance, Budylenko (1978) reported perciform fishes, *Lepidotus caudatus*, in the stomachs of sei whales caught in southern Chile (40° S), and *Notothenia ramsayi* in sei whales taken at Burdwood Bank in the South Atlantic (54° S). In addition, Acevedo et al. (2017) reported surface‐feeding of *Sprattus fuegensis* (order Clupeiformes) in the Strait of Magellan (Chile), through direct observations. With respect to myctophid fishes, *Electrona subaspera* and *Protomyctophum normani* have been previously recorded in the stomachs of sei whales captured around South Georgia Island (Weir and Prieto 2024). Our results extend these observations by identifying two additional species in southern Chile. These lanternfishes are small (2–30 cm in length), typically offshore mesopelagic organisms widely distributed from the Arctic to the Antarctic, and characterized by diel vertical migrations toward surface waters at night (Reyes and Hüne 2012). In Chile, *L. parvicauda* and *T. oculeus* have been reported mainly in northern regions (23° S–27° S) (Acuña 1987; Rodríguez‐Graña and Castro 2003; Quesquén Liza and Ayón Dejo 2012) and in offshore waters between the mainland and Easter Island (27° S, 109° W) (Acuña and Cabrera 2007). Their detection here extends their known distribution to southern Chile (46°–51° S).

Despite no previous records of salmon consumption by sei whales, salmon (Salmonidae) taxa were detected in the Puerto Williams individual. Salmon are common in these waters due to escapes from aquaculture facilities, which are concentrated between 41° and 54° S in Chile, one of the world's leading producers of farmed salmonids (Niklitschek et al. 2013; León‐Muñoz et al. 2018; Quiñones et al. 2019). Salmon hatcheries can provide annual prey subsidies to opportunistic marine predators in British Columbia (Nelson et al. 2019), and humpback whales have been reported to visit hatchery release sites during the liberation of juvenile Chinook salmon ( *Oncorhynchus tshawytscha* ) and coho salmon ( *O. kisutch* ) (Chenoweth et al. 2017). This behavior has not been documented in whales in Chile. However, entanglements of sei whales in salmon farming infrastructure have been reported (Espinosa‐Miranda et al. 2020), indicating spatial overlap between aquaculture and whale foraging areas. Our results suggest that the water filtered by feeding whales contained salmon DNA and/or that sei whales in southern Chile may be consuming escaped and/or naturalized salmon (Sepúlveda et al. 2013; Riva‐Rossi et al. 2025). The detection of prey associated with areas of intensive aquaculture further suggests potential ecological interactions between whale foraging and human activities, warranting further investigation in the context of increasing anthropogenic pressure in the region.

Euphausiids were identified as a fourth prey category but were restricted to the individual from Puerto Williams. Although euphausiids are well‐established components of sei whale diets across ocean basins, such as *Euphausia superba* , *E. vallentini* , *E. lucens* , and *E. frigida* in Antarctic and sub‐Antarctic waters, *E. vallentini* in the South Atlantic and South Pacific (Budylenko 1978; Jackson et al. 2022; Weir and Prieto 2024), *E. pacifica* , *Thysanoessa inermis* , *T. longipes* , and *T. spinifera* in the North Pacific, and *Meganyctiphanes norvegica* and *T. inermis* in the North Atlantic (Budylenko 1978; Kawamura 1980; Mizroch et al. 1984; Jackson et al. 2022; Weir and Prieto 2024), they represented a minor component in our dataset. This pattern suggests a limited contribution of euphausiids relative to other prey, particularly *Grimothea* spp., and may reflect regional differences in prey availability rather than an inherent feeding preference.

### 4.2 Individual‐Level Patterns

Two of the five individuals analyzed were juveniles stranded during the 2015 mass mortality event in southern Chile (Häussermann et al. 2017). Their diet included *Grimothea* spp., copepods, and myctophid fishes. In contrast, the two individuals stranded in Tierra del Fuego and the Puerto Natales showed a highly specialized signal, with stomach contents dominated exclusively by *Grimothea* spp. The Beagle Channel individual exhibited the greatest dietary diversity and was the only whale in which euphausiids were detected. These inter‐individual differences suggest flexible and opportunistic foraging behavior, likely driven by local prey availability and energetic trade‐offs, as described for other balaenopterids (Hildebrand et al. 2022). Similar variability has been reported in fin whales ( *Balaenoptera physalus* ), where individuals may exhibit either specialization on krill or pelagic fish (capelin/herring), or more generalist feeding strategies (Jory et al. 2021).

While DNA metabarcoding enables species‐level identification of prey taxa with greater precision than traditional microscopic examinations (Jeanniard‐du‐Dot et al. 2017; Reidy et al. 2022), including taxa lacking identifiable hard parts (De Vos et al. 2018), sequence read counts should be interpreted with caution. Read abundance can be influenced by predator size, prey digestibility and size, differential amplification efficiency (Deagle et al. 2010; Pompanon et al. 2012) and thus does not necessarily reflect the relative contribution of prey to the diet (Deagle et al. 2019). This limitation may partly explain the absence of prey previously reported for Southern Hemisphere sei whales, such as the amphipod *Themisto gaudichaudii* or small cephalopods (Kawamura 1974; Jackson et al. 2022; Budylenko 1978; Weir and Prieto 2024). In this context, prey prevalence (i.e., the proportion of samples containing a given prey category) has been proposed as a more robust indicator of dietary importance for whales (Reidy et al. 2022). Accordingly, *Grimothea* spp. emerged as the dominant prey category in our dataset, both in terms of prevalence and read abundance.

### 4.3 Potential Novel Parasite–Host Associations in Sei Whales

*Homalometron* corresponds to a genus of trematode parasites that use invertebrates or small fish as intermediate hosts and subsequently infect marine or freshwater fish as their definitive hosts (Stunkard 1964). Our results would suggest that (i) *Homalometron* cercariae were present in the water column filtered by the whales during feeding, prior to infecting their intermediate host and/or (ii) one of the prey items consumed by the whales was parasitized by *Homalometron*. This genus has been reported in fishes from Argentina, with *Homalometron papilliferum* occurring in *Percichthys trucha* (Viozzi et al. 2023). Although there are no records of *Homalometron* in Chile, *P. trucha* is present along the coast associated with rivers and lakes, suggesting that *Homalometron* may be present despite the lack of documented occurrences.

Regarding cestodes, cetaceans become infected with both adult and larval tapeworms, with the adult forms belonging to two families: Tetrabothriidae and Diphyllobothriidae. In whales, Tetrabothriidae are represented by the genera *Priapocephalus* and *Tetrabothrius*. In general, tapeworms of marine homeotherms use zooplanktonic crustaceans as first intermediate hosts. In some tetrabothriids, euphausiids serve as intermediate hosts and fishes as paratenic hosts. In contrast, the life cycles of Diphyllobothriidae involve copepods and fishes as intermediate hosts (Raga et al. 2009). *Tetrabothrius* species are common intestinal parasites in cetaceans, with *Tetrabothrius* spp. reported in a sei whale stranded in Tierra del Fuego (Dougnac 2011), *Tetrabothrius affinis* in sei whales from Argentine Patagonia (Leonardi et al. 2011), in fin whales (Delyamure 1955), and in blue whales in Antarctica (Baylis 1932). Within the genus *Adenocephalus* (family Diphyllobothriidae), *A. pacificus* is considered the most common cestode species in South America and is known to cause fish‐borne diseases (Kuchta et al. 2015). *A. pacificus* is a frequent parasite of marine mammals; however, its presence in whales is poorly documented, with most records occurring in seals and sea lions (Kuchta et al. 2015; Jacobus et al. 2016). Its recent identification in common dolphins ( *Delphinus delphis* ) (Hernández‐Orts et al. 2021) and detection in several individuals in this study suggest that sei whales may act as previously unrecognized hosts or transient carriers within its life cycle.

Parasitological knowledge of Chilean cetaceans remains limited and largely focused on odontocetes. Reported taxa include anisakid nematodes (*Pseudoterranova* spp., *Polymorphus cetaceum* ) and trematodes ( *Synthesium tursionis* ) in the Chilean dolphin ( *Cephalorhynchus eutropia* ) and the Burmeister's porpoise ( *Phocoena spinipinnis* ), as well as *Braunina cordiformis* in the Chilean dolphin and *Anisakis simplex* in the Burmeister's porpoise (Torres et al. 1992). In addition, Alvarado‐Rybak et al. (2020) reported nematode parasites (*Anisakis* spp., *Pseudalius inflexus*, *Stenurus australis*) in Burmeister's porpoises, and *Anisakis* spp. in the common dolphin ( *Delphinus delphis* ) and the pygmy sperm whale ( *Kogia sima* ).

Few cases of protozoan parasites have been reported in marine mammals, and most described species have only recently been identified (Raga and Gulland 2008; Raga et al. 2009). In this study, three families of protozoan parasites (Lecudinidae, Cryptosporidiidae, and Eimeriidae) were detected in the digestive tract of the analyzed sei whales. However, members of Lecudinidae are parasitic protozoans that primarily infect marine invertebrates such as polychaetes, tunicates, mollusks, and crustaceans, with no records in marine mammals (Iritani et al. 2021; Park and Leander 2024). These results may reflect parasites originally infecting polychaetes, tunicates, and/or mollusks that were incidentally ingested by the sei whales, as these taxonomic groups were detected in this study through DNA metabarcoding. Regarding *Cryptosporidium* spp., this parasite has been reported in bowhead whales ( *Balaena mysticetus* ), North Atlantic right whales ( *Eubalaena glacialis* ), and minke whales ( *Balaenoptera acutorostrata* ) (Hughes‐Hanks et al. 2005; Reboredo‐Fernández et al. 2015). Our study expands its known host range to include sei whales, as *Cryptosporidium* spp. was detected in two individuals. Finally, members of the family Eimeriidae are parasites primarily of birds and terrestrial mammals; however, they have been reported in 60 families of marine fishes (Saraiva et al. 2023) but not in cetaceans. Eimeriidae was detected in low read numbers only in the sei whale stranded in Puerto Natales, suggesting they may have been parasites of fish consumed by that whale.

Although gastrointestinal parasites in cetaceans are rarely considered a direct cause of mortality (Geraci and Aubin 1987; Andrade et al. 2001; Striewe et al. 2025), their presence can provide valuable information on health status at both the individual and population levels (Balbuena et al. 1995; Suárez‐González et al. 2024) and from the One Health perspective. Although DNA metabarcoding enables the qualitative characterization of parasite communities at high taxonomic resolution, it has limitations for estimating parasite burden, as the number of sequencing reads obtained does not provide a reliable measure of parasite abundance or infection intensity (Miller et al. 2024).

### 4.4 Limitations of the Study

A small sample size and some methodological limitations should be considered when interpreting the results presented in this work. As with any DNA metabarcoding approach, taxonomic assignments depend on the completeness and accuracy of the reference database. Although we used curated MetaZooGene reference datasets restricted to the South Pacific and South Atlantic to improve the geographic relevance of taxonomic assignments, incomplete representation of regional taxa may still limit taxonomic resolution. This issue may be particularly relevant for parasitic taxa, for which reference sequence coverage can be uneven. Consequently, taxa absent or poorly represented in the reference database may remain unresolved at lower taxonomic ranks or, in some cases, be assigned to closely related represented lineages. Accordingly, fine‐scale taxonomic identifications, particularly those of parasites, should be interpreted with caution, and unresolved assignments were retained at the highest confidently assigned taxonomic level rather than being forced to lower ranks.

It is also important to acknowledge that metabarcoding alone does not always allow for the discrimination between true infections, parasites associated with prey, and/or environmental DNA incidentally present in the digestive tract. Therefore, the interpretation of parasite‐related results should take this methodological limitation into consideration.

Although sample size is very limited, our results align with previous findings on the potential relevance of *Grimothea* spp. as an important prey for sei whales and other marine predators in austral ecosystems (Buchan et al. 2021; Cerchio and Weir 2022; Heredia‐Azuaje et al. 2024; Weir and Prieto 2024; Haro et al. 2025; Andrade et al. 2026). Alternatively, *Grimothea* spp. presence on the digestive tract of sampled individuals might come from secondary consumption given their hard resistant exoskeletons. However, it should be also considered that the size of this species is too large (Pérez‐Barros et al. 2010) and so their potential predators for the latter to be directly consumed by balaenopterids.

Despite these shortcomings, we consider our results relevant considering the limited understanding about sei whale diet composition and parasite loading in the Southern Hemisphere. Moreover, DNA metabarcoding allows the detection of prey that may be difficult or impossible to identify visually and provides relatively fine taxonomic resolution (Pompanon et al. 2012), offering insights into dietary composition that may not be captured by conventional approaches.

## 5 Conclusions

The analysis conducted on five sei whales suggests a previously undocumented trophic reliance on *Grimothea gregaria* in southern Chile, highlighting the importance of regional prey dynamics in shaping sei whale foraging ecology and underscoring the need to incorporate such trophic links into conservation planning. The information reported here contributes to a better understanding of the foraging ecology and parasitology of the endangered sei whale in Patagonia. The results suggest trophic plasticity in sei whales and reinforce the importance of *Grimothea* spp. as a potential key species in austral ecosystems. This study reports information on parasites in sei whales from southern Chile. The report of *Adenocephalus* spp. in sei whales extends the known host range of this genus to large baleen whales, while the presence of *Cryptosporidium* spp. in the species is recorded for the first time. Although DNA metabarcoding is a highly efficient tool, quantitative assessment of diet composition and the understanding of mechanisms behind parasite infestation will require additional complementary techniques.

## Author Contributions

**Daniela Haro:** conceptualization (lead), data curation (equal), funding acquisition (supporting), investigation (lead), project administration (lead), supervision (lead), visualization (equal), writing – original draft (lead), writing – review and editing (equal). **Guillaume Schwob:** conceptualization (equal), data curation (equal), formal analysis (equal), investigation (equal), methodology (lead), resources (equal), software (lead), validation (equal), visualization (equal), writing – review and editing (equal). **Luis Bedriñana‐Romano:** data curation (equal), investigation (equal), writing – review and editing (equal). **Valentina Bernal‐Durán:** data curation (equal), formal analysis (equal), methodology (equal), validation (equal), writing – review and editing (equal). **Benjamín Cáceres:** investigation (equal), writing – review and editing (equal). **Rodrigo Hucke‐Gaete:** funding acquisition (lead), resources (equal), writing – review and editing (equal). **Miguel Cáceres:** funding acquisition (lead), resources (equal), writing – review and editing (equal). **Olivia Blank:** data curation (equal), investigation (equal), writing – review and editing (equal). **Camila Calderón‐Quirgas:** investigation (equal), writing – review and editing (equal). **Mauricio Ulloa:** data curation (equal), writing – review and editing (equal). **Francisco Viddi:** data curation (equal), investigation (equal), writing – review and editing (equal).

## Funding

This work was supported by Agencia Nacional de Investigacion y Desarrollo, Chile, Fondecyt Postdoctoral No. 3210475.

## Conflicts of Interest

The authors declare no conflicts of interest.

## Supporting information


**Figure S1:** Rarefaction curves for the COI and 18S amplicons.
**Table S1:** Denoising and removal of read contaminations in the COI gene libraries generated from stomach and intestine contents of Sei whales using the DADA2 pipeline.
**Table S2:** Denoising and removal of read contaminations in the 18S rRNA gene libraries generated from stomach and intestine contents of Sei whales using the DADA2 pipeline.
**Table S3:** List of taxa, number of ASVs and number of readings obtained with the COI amplicon in five sei whales stranded in southern Chile.
**Table S4:** List of taxa, number of ASVs and number of readings obtained with the 18S amplicon in five sei whales stranded in southern Chile.

## Data Availability

All sequences are deposited in the NCBI Sequence Read Archive under the identification PRJNA1449745.
