# Supplementary material for: Opportunistic Feeding and Parasite Assemblages in Sei Whales (Balaenoptera borealis) From Southern Chile Revealed by DNA Metabarcoding
Source: Ecol Evol. 2026 Sep 28;16(10):e74427. doi: 10.1002/ece3.74427 (PMC13619459; doi:10.1002/ece3.74427)
Supplement: Supplementary file 1 [file ECE3-16-e74427-s001.zip › ece374427-sup-0002-FigureS1-TableS1-S4@Supplementary Material.docx]

**Figure S1**: Rarefaction curves for the COI and 18S amplicons.

**Table S1**: Denoising and removal of read contaminations in the COI gene libraries generated from stomach and intestine contents of Sei whales using the DADA2 pipeline.

| **Samples** | **Input** | **Filtered** | **denoisedF** | **denoisedR** | **Merged** | **Nonchim** | **Post singleton filtering** | **Post taxonomic filtering** |
| --- | --- | --- | --- | --- | --- | --- | --- | --- |
| Total (all reads) | 2167498 | 1656777 | 1649329 | 1649478 | 1619329 | 629675 | 622384 | 609398 |
| Total (blanks) | 20384 | 15605 | 15254 | 15244 | 14713 | 7296 | 5 | 5 |
| Ctrl-NTC1 | 20384 | 15605 | 15254 | 15244 | 14713 | 7296 | 5 | 5 |
| Total (true samples) | 2147114 | 1641172 | 1634075 | 1634234 | 1604616 | 622379 | 622379 | 609393 |
| B1 | 298350 | 223531 | 223289 | 223202 | 222367 | 121957 | 121957 | 121538 |
| B2 | 345743 | 264611 | 264138 | 264113 | 262295 | 119379 | 119379 | 118587 |
| B3 | 351988 | 270375 | 268489 | 268500 | 254600 | 79570 | 79570 | 74017 |
| B21 | 313479 | 239988 | 239677 | 239677 | 238640 | 108158 | 108158 | 107449 |
| B22 | 457835 | 350410 | 349389 | 349251 | 346528 | 130889 | 130889 | 128627 |
| IC B22 | 379719 | 292257 | 289093 | 289491 | 280186 | 62426 | 62426 | 59175 |

**Table S2**: Denoising and removal of read contaminations in the 18S rRNA gene libraries generated from stomach and intestine contents of Sei whales using the DADA2 pipeline.

| **Samples** | **Input** | **Filtered** | **denoisedF** | **denoisedR** | **Merged** | **Nonchim** | **Post singleton filtering** | **Post taxonomic filtering** |
| --- | --- | --- | --- | --- | --- | --- | --- | --- |
| Total (all reads) | 2181103 | 1679872 | 1677508 | 1678169 | 1641816 | 1509748 | 1509746 | 1495905 |
| Total (blanks) | 7 | 6 | 5 | 5 | 5 | 5 | 5 | NA |
| Ctrl-NTC1 | 7 | 6 | 5 | 5 | 5 | 5 | 5 | 5 |
| Total (true samples) | 2181096 | 1679866 | 1677503 | 1678164 | 1641811 | 1509743 | 1509741 | 1495900 |
| B1 | 424396 | 326374 | 325432 | 325937 | 312772 | 223245 | 223245 | 223164 |
| B2 | 366133 | 282554 | 282127 | 282020 | 274896 | 271260 | 271258 | 271003 |
| B3 | 276699 | 212719 | 212643 | 212635 | 211273 | 209797 | 209797 | 242057 |
| B21 | 424638 | 326870 | 326746 | 326736 | 320944 | 287721 | 287721 | 242057 |
| B22 | 294155 | 226355 | 225859 | 226097 | 222224 | 220019 | 220019 | 219963 |
| IC B22 | 395075 | 304994 | 304696 | 304739 | 299702 | 297701 | 297701 | 297656 |

**Table S3**: List of taxa, number of ASVs and number of readings obtained with the COI amplicon in five sei whales stranded in southern Chile.

| **Kingdom** | **Phylum** | **Class** | **Order** | **Family** | **Genus** | **Species** | **ASV** | **B21** | **B22** | **IC B22** | **B1** | **B2** | **B3** |
| --- | --- | --- | --- | --- | --- | --- | --- | --- | --- | --- | --- | --- | --- |
| Animalia | - | - | - | - | - | - | 1038 | 1301 | 5548 | 13217 | 919 | 3194 | 1370 |
| Animalia | Rotifera | Bdelloidea | Philodinida | Philodinidae | - | *-* | 38 | 154 | 299 | 379 | 41 | 398 | 3138 |
| Animalia | Porifera | Demospongiae | - | - | - | *-* | 1 | 0 | 0 | 16 | 0 | 0 | 0 |
| Animalia | Cnidaria | - | - | - | - | *-* | 24 | 13 | 119 | 435 | 71 | 0 | 99 |
| Animalia | Cnidaria | Hydrozoa | - | - | - | *-* | 54 | 0 | 297 | 902 | 31 | 201 | 697 |
| Animalia | Cnidaria | Hydrozoa | Siphonophorae | - | - | *-* | 1 | 0 | 0 | 0 | 11 | 0 | 0 |
| Animalia | Annelida | Polychaeta | Capitellida | Capitellidae | - | *-* | 24 | 0 | 0 | 267 | 0 | 37 | 322 |
| Animalia | Annelida | Polychaeta | Phyllodocida | Tomopteridae | Tomopteris |  | 9 | 0 | 0 | 0 | 0 | 0 | 389 |
| Animalia | Mollusca | - | - | - | - | *-* | 1 | 0 | 0 | 167 | 0 | 0 | 0 |
| Animalia | Mollusca | Gastropoda | Stylommatophora | Limacinidae | Limacina | *-* | 10 | 32 | 0 | 0 | 0 | 0 | 533 |
| Animalia | Arthropoda | Copepoda | Calanoida | Calanidae | Calanus | *Calanus chilensis* | 7 | 0 | 0 | 0 | 0 | 0 | 534 |
| Animalia | Arthropoda | Copepoda | Calanoida | Subeucalanidae | *Subeucalanus* | *-* | 6 | 110 | 186 | 0 | 0 | 0 | 95 |
| Animalia | Arthropoda | Copepoda | Calanoida | Eucalanidae | *Eucalanus* | *-* | 1 | 110 | 0 | 0 | 0 | 0 | 0 |
| Animalia | Arthropoda | Copepoda | Calanoida | - | - |  | 2 | 0 | 107 | 0 | 0 | 0 | 0 |
| Animalia | Arthropoda | Malacostraca | Decapoda | Munididae | Grimothea | *Grimothea gregaria* | 10 | 0 | 162 | 0 | 0 | 0 | 344 |
| Animalia | Arthropoda | Malacostraca | Euphausiacea | Euphausiidae | Euphausia | *-* | 10 | 0 | 0 | 0 | 0 | 0 | 308 |
| Animalia | Arthropoda | Malacostraca | - | - | - | *-* | 5 | 0 | 98 | 106 | 0 | 64 | 186 |
| Animalia | Arthropoda | - | - | - | - | *-* | 4 | 0 | 0 | 88 | 0 | 0 | 87 |
| Animalia | Arthropoda | Ostracoda | Halocyprida | - | - | *-* | 2 | 46 | 0 | 0 | 0 | 92 | 0 |
| Animalia | Platyhelminthes | - | - | - | - | *-* | 2 | 0 | 0 | 284 | 0 | 0 | 0 |
| Animalia | Chaetognatha | Sagittoidea | - | - | - | *-* | 1 | 0 | 0 | 0 | 0 | 0 | 70 |
| Animalia | Chaetognatha | Sagittoidea | Aphragmophora | Sagittidae | Flaccisagitta | *-* | 12 | 0 | 0 | 0 | 43 | 151 | 345 |
| Animalia | Chaetognatha | Sagittoidea | Aphragmophora | Sagittidae | Pseudosagitta | *-* | 7 | 0 | 147 | 0 | 65 | 0 | 426 |
| Animalia | Chaetognatha | Sagittoidea | Aphragmophora | Sagittidae | - | *-* | 2 | 0 | 0 | 0 | 0 | 0 | 57 |
| Animalia | Echinodermata | - | - | - | - | *-* | 1 | 0 | 28 | 0 | 0 | 0 | 0 |
| Animalia | Chordata | - | - | - | - | *-* | 3 | 0 | 22 | 41 | 0 | 0 | 34 |
| Animalia | Chordata | **Actinopterygii** | - | - | - | *-* | 11 | 0 | 12 | 0 | 0 | 0 | 440 |
| Animalia | Chordata | **Actinopterygii** | Myctophiformes | Myctophidae | Triphoturus | *Triphoturus oculeum* | 4 | 0 | 0 | 185 | 0 | 0 | 0 |
| Animalia | Chordata | **Actinopterygii** | Myctophiformes | Myctophidae | Lampanyctus | *Lampanyctus parvicauda* | 2 | 0 | 0 | 0 | 0 | 373 | 0 |
| Animalia | Chordata | **Actinopterygii** | Salmoniformes | Salmonidae | Salmo | *-* | 1 | 0 | 0 | 0 | 0 | 93 | 0 |
| Animalia | Chordata | Mammalia | Artiodactyla | Balaenopteridae | Balaenoptera | *Balaenoptera borealis* | 85 | 105793 | 121418 | 43101 | 120288 | 113952 | 51075 |
| Plantae | Rhodophyta | Florideophyceae | - | - | - | *-* | 27 | 0 | 331 | 27 | 134 | 183 | 1467 |

**Table S4:** List of taxa, number of ASVs and number of readings obtained with the 18S amplicon in five sei whales stranded in southern Chile.

| **Kingdom** | **Phylum** | **Class** | **Order** | **Family** | **Genus** | **ASV** | **B21** | **B22** | **IC B22** | **B1** | **B2** | **B3** |
| --- | --- | --- | --- | --- | --- | --- | --- | --- | --- | --- | --- | --- |
| Animalia | - | - | - | - | - | 7 | 3 | 0 | 0 | 0 | 42 | 0 |
| Animalia | Cnidaria | Hydrozoa | Siphonophorae | Clausophyidae | Crystallophyes | 2 | 0 | 0 | 0 | 0 | 212 | 0 |
| Animalia | Cnidaria | Hydrozoa | Narcomedusae | Solmundaeginidae | Solmundella | 1 | 0 | 0 | 0 | 0 | 6 | 0 |
| Animalia | Cnidaria | Scyphozoa | Semaeostomeae | Ulmaridae | - | 1 | 0 | 0 | 0 | 0 | 83 | 0 |
| Animalia | Ctenophora | Nuda | Beroida | Beroidae | Beroe | 1 | 0 | 0 | 0 | 0 | 4 | 0 |
| Animalia | Mollusca | Gastropoda | Pteropoda | Limacinoidea | Limacina | 1 | 0 | 0 | 0 | 0 | 21 | 0 |
| Animalia | Echinodermata | Echinoidea | Camarodonta | Temnopleuridae | Pseudechinus | 1 | 0 | 0 | 0 | 0 | 16 | 0 |
| Animalia | Arthropoda | - | - | - | - | 1 | 312 | 0 | 0 | 0 | 0 | 6 |
| Animalia | Arthropoda | Malacostraca | Decapoda | Munididae | Grimothea | 4 | 199 | 144 | 102 | 134 | 129 | 86695 |
| Animalia | Arthropoda | Malacostraca | Euphausiacea | Euphausiidae | Euphausia | 1 | 0 | 0 | 0 | 0 | 6 | 0 |
| Animalia | Arthropoda | Copepoda | Calanoida | Calanidae | Calanoides | 1 | 0 | 0 | 0 | 0 | 61 | 0 |
| Animalia | Arthropoda | Copepoda | Calanoida | Metridinidae | Metridia | 1 | 0 | 0 | 0 | 0 | 18 | 0 |
| Animalia | Arthropoda | Copepoda | Calanoida | Clausocalanidae | Ctenocalanus | 1 | 0 | 0 | 0 | 0 | 13 | 0 |
| Animalia | Arthropoda | Copepoda | Cyclopoida | Oithonidae | Oithona | 1 | 0 | 0 | 0 | 0 | 8 | 0 |
| Animalia | Chordata | - | - | - | - | 14 | 212948 | 359 | 378 | 473 | 323 | 64717 |
| Animalia | Chordata | Ascidiacea | Enterogona | Perophoridae | - | 9 | 4272 | 4 | 2 | 20 | 48 | 3 |
| Animalia | Chordata | Thaliacea | Salpida | Salpinae | Ihlea | 2 | 0 | 9 | 0 | 0 | 10 | 0 |
| Animalia | Platyhelminthes | - | - | - | - | 1 | 0 | 0 | 6 | 0 | 0 | 0 |
| Animalia | Platyhelminthes | Trematoda | - | - | - | 5 | 0 | 0 | 0 | 1790 | 49 | 0 |
| Animalia | Platyhelminthes | Trematoda | Plagiorchiida | - | - | 51 | 1594 | 5781 | 8410 | 152136 | 257204 | 765 |
| Animalia | Platyhelminthes | Trematoda | Plagiorchiida | Apocreadiidae | - | 6 | 0 | 16 | 0 | 1165 | 135 | 0 |
| Animalia | Platyhelminthes | Trematoda | Plagiorchiida | Apocreadiidae | Homalometron | 212 | 6219 | 212962 | 288534 | 67102 | 8380 | 1240 |
| Animalia | Platyhelminthes | Cestoda | Tetrabothriidea | Tetrabothriidae | Tetrabothrius | 1 | 9 | 7 | 11 | 206 | 9 | 4644 |
| Animalia | Platyhelminthes | Cestoda | Diphyllobothriidea | Diphyllobothriidae | Adenocephalus | 1 | 0 | 553 | 97 | 4 | 0 | 4 |
| Animalia | Platyhelminthes | Rhabditophora | Proseriata | Otoplanidae | - | 1 | 0 | 0 | 0 | 0 | 23 | 0 |
| Chromista | - | - | - | - | - | 9 | 0 | 0 | 0 | 0 | 129 | 0 |
| Chromista | Dinoflagellata | - | - | - | - | 1 | 0 | 0 | 0 | 0 | 6 | 0 |
| Chromista | Dinoflagellata | Dinophyceae | - | - | - | 4 | 0 | 0 | 4 | 0 | 41 | 0 |
| Chromista | Dinoflagellata | Dinophyceae | Gymnodiniales | Gymnodiniaceae | Gyrodinium | 3 | 0 | 0 | 0 | 0 | 159 | 0 |
| Chromista | Dinoflagellata | Dinophyceae | Gymnodiniales | Gymnodiniaceae | - | 1 | 0 | 0 | 0 | 0 | 14 | 0 |
| Chromista | Dinoflagellata | Dinophyceae | Gymnodiniales | Warnowiaceae | Warnowia | 2 | 0 | 0 | 0 | 0 | 41 | 0 |
| Chromista | Dinoflagellata | Dinophyceae | Gymnodiniales | Kareniaceae | - | 1 | 0 | 0 | 0 | 0 | 11 | 0 |
| Chromista | Dinoflagellata | Dinophyceae | Gymnodiniales | - | - | 3 | 0 | 21 | 0 | 0 | 52 | 0 |
| Chromista | Dinoflagellata | Dinophyceae | Suessiales | Suessiaceae | - | 1 | 0 | 0 | 0 | 0 | 20 | 0 |
| Chromista | Dinoflagellata | Dinophyceae | Peridiniales | Peridiniaceae | Peridinium | 1 | 0 | 0 | 0 | 0 | 41 | 0 |
| Chromista | Dinoflagellata | Dinophyceae | Peridiniales | Peridiniaceae | Kryptoperidinium | 1 | 0 | 0 | 0 | 0 | 21 | 0 |
| Chromista | Dinoflagellata | Dinophyceae | Peridiniales | Protoperidiniaceae | Protoperidinium | 1 | 0 | 0 | 0 | 0 | 14 | 0 |
| Chromista | Dinoflagellata | Dinophyceae | Peridiniales | Peridiniaceae | Scrippsiella | 1 | 0 | 0 | 0 | 0 | 167 | 0 |
| Chromista | Dinoflagellata | Dinophyceae | Gonyaulacales | Goniodomataceae | - | 2 | 0 | 0 | 0 | 0 | 52 | 0 |
| Chromista | Bacillariophyta | Bacillariophyceae | Naviculales | Naviculaceae | - | 1 | 0 | 0 | 0 | 0 | 152 | 0 |
| Chromista | Bacillariophyta | Bacillariophyceae | Bacillariales | Fragilariopsis | - | 1 | 0 | 0 | 0 | 0 | 6 | 0 |
| Chromista | Bacillariophyta | Bacillariophyceae | Rhizosoleniales | Rhizosoleniaceae | Proboscia | 1 | 0 | 0 | 0 | 0 | 6 | 0 |
| Chromista | Bacillariophyta | Bacillariophyceae | Triceratiales | Triceratiaceae | Eupodiscus | 1 | 0 | 0 | 0 | 0 | 25 | 0 |
| Chromista | Bacillariophyta | Bacillariophyceae | Achnanthales | Achnanthaceae | Achnanthes | 1 | 0 | 0 | 0 | 0 | 44 | 0 |
| Chromista | Haptophyta | Coccolithophyceae | Phaeocystales | Phaeocystaceae | Phaeocystis | 1 | 0 | 0 | 0 | 0 | 151 | 0 |
| Chromista | Ochrophyta | Chrysophyceae | Chromulinales | - | - | 1 | 0 | 0 | 0 | 0 | 15 | 0 |
| Chromista | Ochrophyta | Raphidophyceae | - | - | - | 3 | 0 | 0 | 0 | 0 | 30 | 0 |
| Chromista | Ochrophyta | Dictyochophyceae | Pedinellales | Pedinellaceae | Ciliophrys | 2 | 0 | 0 | 0 | 0 | 120 | 0 |
| Chromista | Ochrophyta | Phaeophyceae | Laminariales | Alariaceae | Undaria | 10 | 0 | 0 | 79 | 77 | 0 | 0 |
| Chromista | Ochrophyta | Phaeophyceae | Laminariales | Laminariaceae | - | 1 | 0 | 0 | 0 | 0 | 4 | 0 |
| Chromista | Cryptophyta | Cryptophyceae | Pyrenomonadales | Geminigeraceae | Teleaulax | 1 | 0 | 0 | 0 | 0 | 4 | 0 |
| Chromista | Cryptophyta | - | - | - | - | 1 | 0 | 0 | 0 | 0 | 34 | 0 |
| Protozoa | - | - | - | - | - | 20 | 0 | 0 | 2 | 0 | 402 | 0 |
| Protozoa | Bigyra | Labyrinthulea | Thraustochytrida | Aplanochytriidae | Aplanochytrium | 1 | 0 | 0 | 0 | 0 | 4 | 0 |
| Protozoa | Oomycota | Peronosporea | - | - | - | 1 | 0 | 0 | 0 | 0 | 7 | 0 |
| Protozoa | Ciliophora | - | - | - | - | 2 | 0 | 0 | 0 | 0 | 16 | 0 |
| Protozoa | Ciliophora | Oligotrichea | - | - | - | 2 | 0 | 0 | 0 | 0 | 89 | 0 |
| Protozoa | Ciliophora | Oligotrichea | Oligotrichida | Strombidiidae | Strombidium | 8 | 19 | 8 | 9 | 12 | 675 | 6477 |
| Protozoa | Ciliophora | Oligotrichea | Choreotrichida | Strobilidiidae | Pelagostrobilidium | 1 | 0 | 0 | 0 | 0 | 14 | 0 |
| Protozoa | Ciliophora | Oligotrichea | Choreotrichida | Strombidinopsidae | Strombidinopsis | 1 | 0 | 0 | 0 | 0 | 19 | 0 |
| Protozoa | Ciliophora | Oligotrichea | Oligotrichida | Tontoniidae | Pseudotontonia | 1 | 0 | 0 | 0 | 0 | 10 | 0 |
| Protozoa | Ciliophora | Heterotrichea | Heterotrichida | Condylostomatidae | Condylostoma | 4 | 0 | 0 | 0 | 0 | 704 | 0 |
| Protozoa | Ciliophora | Spirotrichea | Hypotrichia | Holostichidae | Holosticha | 1 | 0 | 0 | 0 | 0 | 15 | 0 |
| Protozoa | Ciliophora | Oligohymenophorea | Philasterida | Parauronematidae | Miamiensis | 1 | 0 | 0 | 0 | 0 | 11 | 0 |
| Protozoa | Ciliophora | Litostomatea | Pleurostomatida | Litonotidae | - | 3 | 0 | 0 | 0 | 0 | 12 | 0 |
| Protozoa | Cercozoa | Imbricatea | Marimonadida | - | - | 2 | 0 | 0 | 0 | 0 | 18 | 0 |
| Protozoa | Cercozoa | Imbricatea | Euglyphida | - | - | 1 | 0 | 0 | 0 | 0 | 7 | 0 |
| Protozoa | Cercozoa | Imbricatea | - | - | - | 1 | 0 | 0 | 0 | 0 | 6 | 0 |
| Protozoa | Cercozoa | Chlorarachnea | Chlorachniida | Chlorarachniaceae | Bigelowiella | 1 | 0 | 0 | 0 | 0 | 6 | 0 |
| Protozoa | Cercozoa | Granofilosea | Leucodictyida | Massisteriidae | Massisteria | 1 | 0 | 0 | 0 | 0 | 18 | 0 |
| Protozoa | Apicomplexa | Conoidasida | Eugregarinorida | Lecudinidae | - | 1 | 28 | 8 | 21 | 10 | 15 | 8954 |
| Protozoa | Apicomplexa | Conoidasida | Eucoccidiorida | Cryptosporidiidae | Cryptosporidium | 3 | 0 | 0 | 4 | 0 | 35 | 0 |
| Protozoa | Apicomplexa | Conoidasida | Eucoccidiorida | Eimeriidae | - | 1 | 0 | 0 | 0 | 0 | 27 | 0 |
| Protozoa | Euglenozoa | Kinetoplastea | Eubodonida | - | - | 1 | 0 | 0 | 0 | 0 | 5 | 0 |
| Protozoa | Choanozoa | Choanoflagellatea | Craspedida | Salpingoecaceae | - | 3 | 0 | 0 | 0 | 0 | 33 | 0 |
| Protozoa | Choanozoa | Choanoflagellatea | Acanthoecida | - | - | 3 | 0 | 0 | 0 | 0 | 184 | 0 |
| Protozoa | Choanozoa | - | - | - | - | 1 | 0 | 0 | 0 | 0 | 64 | 0 |
| Protozoa | Amoebozoa | Discosea | Dactylopodida | Paramoebidae | - | 1 | 0 | 0 | 0 | 0 | 3 | 0 |
| Plantae | Tracheophyta | Magnoliopsida | Malpighiales | Euphorbiaceae | - | 3 | 3374 | 0 | 0 | 0 | 0 | 0 |
| Plantae | Tracheophyta | Magnoliopsida | Lamiales | Acanthaceae | - | 3 | 3696 | 0 | 0 | 0 | 0 | 0 |
| Plantae | Tracheophyta | Magnoliopsida | - | - | - | 2 | 0 | 0 | 0 | 14 | 12 | 0 |
| Plantae | Chlorophyta | Pyramimonadophyceae | Pseudoscourfieldiales | Pycnococcaceae | Pseudoscourfieldia | 1 | 21 | 10 | 10 | 18 | 7 | 5866 |
| Plantae | Chlorophyta | Pyramimonadophyceae | Pyramimonadales | Pyramimonadaceae | Pyramimonas | 1 | 0 | 0 | 0 | 0 | 7 | 0 |
| Plantae | Chlorophyta | Ulvophyceae | Ulotrichales | Ulotrichaceae | - | 2 | 0 | 0 | 0 | 0 | 28 | 0 |
| Plantae | Chlorophyta | Ulvophyceae | Ulvales | Kornmanniaceae | Pseudendoclonium | 1 | 0 | 0 | 0 | 0 | 12 | 0 |
| Plantae | Chlorophyta | Ulvophyceae | Ulvales | Ulvellaceae | Ulvella | 1 | 0 | 0 | 0 | 0 | 11 | 0 |
| Plantae | Chlorophyta | Ulvophyceae | Ulvales | Gayraliaceae | Gayralia | 1 | 0 | 0 | 0 | 0 | 18 | 0 |
| Plantae | Chlorophyta | Trebouxiophyceae | Prasiolales | Prasiolaceae | Prasiola | 1 | 0 | 0 | 0 | 0 | 9 | 0 |
| Plantae | Chlorophyta | - | - | - | - | 1 | 0 | 0 | 0 | 0 | 10 | 0 |
